# Comparison Between Signal Transduction Pathway Activity in Blood Cells of Sepsis Patients and Laboratory Models

**DOI:** 10.3390/cells14040311

**Published:** 2025-02-19

**Authors:** Wilbert Bouwman, Reinier Raymakers, Tom van der Poll, Anja van de Stolpe

**Affiliations:** 1 Center of Experimental and Molecular Medicine & Division of Infectious Diseases, Amsterdam University Medical Center, University of Amsterdam, 1105 AZ Amsterdam, The Netherlands; 2 DCDC-Tx B.V., 5263 EM Vught, The Netherlands

**Keywords:** sepsis, LPS, signal transduction pathways, quantitative assessment, immune response, innate and adaptive immune system, disease model

## Abstract

Sepsis represents a serious disease burden that lacks effective treatment. Drug development for sepsis requires laboratory models that adequately represent sepsis patients. Simultaneous Transcriptome-based Activity Profiling of Signal Transduction Pathway (STAP-STP) technology quantitatively infers STP activity from mRNA levels of target genes of the STP-associated transcription factor. Here, we used STAP-STP technology to compare STP activities between sepsis patients and lipopolysaccharide (LPS)-based models. Activity scores of Androgen Receptor (AR), TGFβ, NFκB, JAK-STAT1/2, and JAK-STAT3 STPs were calculated based on publicly available transcriptome data. Peripheral blood mononuclear cells (PBMCs) from patients with Gram-negative sepsis, nor PBMCs stimulated with LPS in vitro, showed altered STP activity. Increased NFκB, JAK-STAT1/2, and JAK-STAT3 STP activity was found in whole blood stimulated with LPS in vitro, and in whole blood obtained after intravenous injection of LPS in humans in vivo; AR and TGFβ STP activity only increased in the in vivo LPS model. These results resembled previously reported STP activity in whole blood of sepsis patients. We provide the first comparison of STP activity between patients with sepsis and laboratory model systems. Results are of use for the refinement of sepsis model systems for rational drug development.

## 1. Introduction

Sepsis is a life-threatening infection in which the immune response is dysregulated, resulting in multi-organ dysfunction or failure as the leading cause of sepsis-related deaths [1]. The global burden of sepsis is tremendous, with an estimated 48.9 million incident cases and 11.0 million deaths in 2017, representing nearly 20% of all deaths worldwide [2]. Sepsis is generally caused by an uncontrolled bacterial infection and is characterized by a systemic inflammatory response [1,3]. Aside from antibiotics and supportive measures, no treatments have been shown to improve clinical outcomes [3,4]. Developing effective treatments is complicated by heterogeneity in the host response due to multiple factors, including age, comorbidities, genetic background, the source of infection, and the causative pathogen [5,6]. Pathogen-associated molecular patterns, particularly lipopolysaccharide (LPS) derived from Gram-negative bacteria, induce systemic inflammation that mimics many of the initial clinical features of sepsis [7,8]. LPS is therefore a frequently used agonist to study inflammatory responses in laboratory model systems relevant for sepsis [7,8]. Representative models that sufficiently mimic alterations in immune cell function found in patients with sepsis, are necessary to discover drugs to treat sepsis [9].

Cell function is controlled by a limited set of cellular signal transduction pathways (STPs) [10]. RNA-based computational analysis methods have been developed to quantitatively measure the activity of clinically relevant STPs in cell and tissue samples [11,12,13,14,15,16], including blood and immune cells [12,17,18,19]. This Simultaneous Transcriptome-based Activity Profiling of Signal Transduction Pathway (STAP-STP) technology makes it possible to directly and quantitatively compare STP activity in patients with sepsis and in sepsis model systems. Unlike publicly available transcriptome analysis tools, our STP analysis models have been extensively validated for their capability to accurately infer quantitative activity of STPs from transcriptome data of individual samples [19]. This offers completely novel insights into sepsis laboratory models and the pathophysiology of clinical sepsis. This comparison is of use for drug development as it can guide future discovery of drugs that can correct aberrant STP activity in immune cells [19].

We reported earlier on aberrant STP activity in whole blood of sepsis patients, using publicly available transcriptomics data [17]. In the present study, we compared STP analysis results between clinical sepsis studies and LPS-based in vitro and in vivo models, using RNA expression profiles derived from whole blood or peripheral blood mononuclear cells (PBMCs).

## 2. Materials and Methods

### 2.1. Measurement of Activity of Signal Transduction Pathways

mRNA-based assays for measuring the activity of the Nuclear Factor kappa B (NFκB), Androgen Receptor (AR), Transforming Growth Factor beta (TGFβ), Janus Kinase (JAK)-Signal Transducer, Activator of Transcription (STAT) 1/2, and JAK-STAT3 STPs have been developed and validated, and can be used to analyze STP activity on any cell type [11,12,13,14,15,16], including blood cells [12,17,18,19]. AR [20], TGFβ [21], NFκB [22], JAK-STAT1/2 [23], and JAK-STAT3 [24] were selected in this study based on their known role in the immune response and during infection [12,16,17,25]. For each STP, activity is measured by a Bayesian modeling-based probabilistic computational model that infers an STP activity score from expression levels of a defined set of 20–30 mRNAs transcribed from target genes of the STP-associated transcription factor (Supplementary Figure S1, for target genes and STP model validation, see for AR, TGFβ, NFκB [14] and JAK-STAT1/2 and JAK-STAT3 [12]). STP activity scores are highly reproducible and quantitative [13,25]. For the current study, pathway activity scores (PAS) were measured using publicly available Affymetrix expression microarray data (Affymetrix HG-U133Plus2.0 microarray platform) derived from the GEO database (www.ncbi.nlm.nih.gov/geo (accessed on 1 September 2024). Included datasets consist of patients with sepsis and laboratory model systems with relevance for sepsis (see below). Quantitative log2 odds scores for STP activity were calculated as described [11,13,14].

### 2.2. Microarray Data Quality Control

Quality control (QC) was performed on Affymetrix data of each sample as described [26]. QC parameters included: the average value of all probe intensities, presence of negative or extremely high (>16-bit), intensity values, poly-A RNA (sample preparation spike-ins) and labeled cRNA (hybridization spike-ins) controls, GAPDH and ACTB 3′/5′ ratio, center of intensity, and values of positive and negative border controls. QC parameters were determined by the affyQCReport package in R, and RNA degradation value was determined by the AffyRNAdeg function from the Affymetrix package in R [26,27].

### 2.3. GEO Datasets

Analyzed in vitro and in vivo LPS-based models and clinical studies are described below. For full descriptions and details, see study-associated cited articles.

#### 2.3.1. Study on PBMCs of Sepsis Patients

A. GSE9960 [28]: PBMCs isolated from healthy subjects and sepsis patients infected by Gram-positive bacteriae, Gram-negative bacteriae or both, or by an unidentified microorganism. For patient characteristics see Supplementary Materials Figure S2.

#### 2.3.2. Studies on In Vitro LPS-Stimulated Whole Blood or PBMC Samples

B. GSE20114 [29]: Whole blood from hypertriglyceridemic men stimulated in vitro for 4 h with 10 µg/mL LPS (Escherichia coli O157:H7) or vehicle. Docosahexaenoic acid (DHA) treatment groups were excluded from the analysis.
C. GSE46914 [30]: PBMCs from healthy donors stimulated once with LPS (100 ng/mL; LPS unprimed) or twice (LPS primed). To induce the LPS-primed state, PBMCs were first cultured in the presence or absence (control group) of 2 ng/mL LPS (mix from *Escherichia coli* O55:B5, O127:B8, and O111:B4) for 15 h. Then, PBMCs were washed, left untreated for 24 h, and subsequently stimulated with LPS (100 ng/mL) for 6 h. LPS priming is used to induce LPS tolerance that mimics immunosuppression. LPS tolerance can be induced by exposure to low concentrations of LPS.

#### 2.3.3. In Vivo Human Endotoxemia Whole Blood Model Study

D. GSE3284 [31]: Human endotoxemia model. Intravenous administration of LPS (2 ng/kg body weight) to healthy humans. Whole blood was collected before LPS administration and 2 and 6 h thereafter for leukocyte isolation.

### 2.4. Comparison of STP Analysis Results with Results Described in Dataset-Associated Publications

STAP-STP analysis results were compared with the transcriptome analysis results reported in the original publication associated with the public datasets [28,29,30,31]. In these publications, publicly available transcriptome analysis tools were used to analyze transcriptome data. In the Supplementary Materials, a comparison with STAP-STP analysis results is summarized.

### 2.5. General Rules for Interpretation of STP Activity Score

An important and unique advantage of the STP activity assays lies in their applicability to every cell type. However, certain factors should be taken into account when interpreting log2 odds STP activity scores, as previously explained in [14].

(1) In the same sample, log2 odds STP activity scores cannot be compared quantitatively between different STPs, since each STP has its own range (ranging from minimum to maximum activity) in log2 odds activity scores.
(2) The log2 odds range for STP activity may vary depending on cell type. Once the range has been defined by using samples with known pathway activity, subsequent samples can be directly interpreted against this reference, allowing for the assessment of absolute values. In the absence of a defined range, only differences in log2 odds activity score between samples can be interpreted. For each dataset, STP activity scores are plotted on the log2 odds scale.
(3) STP activity scores are highly quantitative; small differences in log2 odds PAS can be reproducible and meaningful [13,25].
(4) A negative log2 odds ratio does not necessarily mean that the pathway is inactive (see 2: for a specific cell type, the activity range may for example, lie between −20 log2 odds for an inactive STP to −5 log2 odds for a maximal active STP).

### 2.6. Statistics

Boxplots and individual sample plots are overlaid in figures and were made using the Python (version 3) data visualization library function ‘seaborn’; dots represent the individual samples. Additional statistical annotations were created using the Python package ’statannot’. Linear mixed effects model *p*-value calculations were made in R. For datasets GSE9960 and GSE46914, a two-sided Mann–Whitney *t*-test with Bonferroni correction was applied to compare PAS across groups within the dataset. For dataset GSE20114, a two-sided paired *t*-test was applied, while for dataset GSE3284, a linear mixed effects model was applied to compare PAS across groups within the dataset. *p* < 0.01 was considered to represent a statistically significant difference.

## 3. Results

To assess which sepsis model is most representative for patients with Gram-negative sepsis, we compared STP activity profiles between clinical sepsis patients and available LPS-based sepsis models (PBMCs and whole blood).

### 3.1. STP Activity in PBMCs from Patients with Sepsis

We previously reported increased AR and TGFβ STP activity (and a trend towards higher NFκB and JAK-STAT3 STP activity) in whole blood of adult and pediatric sepsis patients [17]. The current analysis of transcriptome data from PBMCs of sepsis patients enabled comparison between the STP profile in whole blood and PBMCs [28].

**Figure 1:**
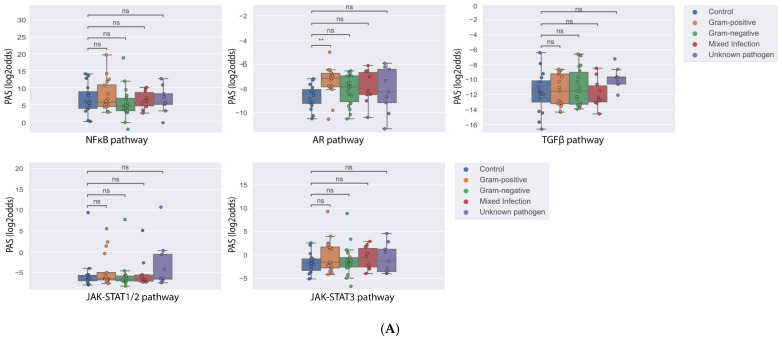

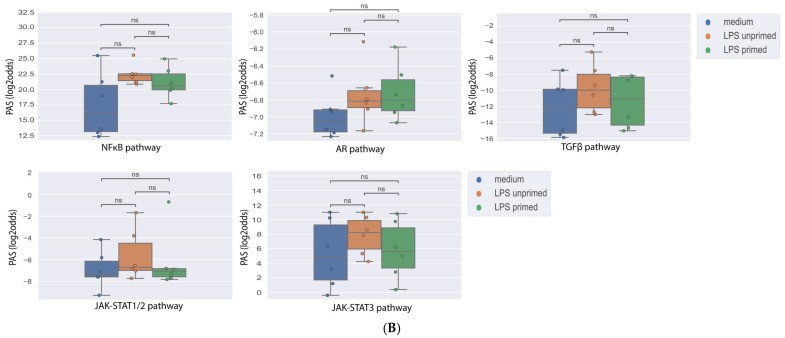
Pathway activity scores of PBMCs from sepsis patients and after stimulation with LPS in vitro. (**A**) GSE9960 [28]: PBMCs isolated from healthy and sepsis patients infected with Gram-positive, Gram-negative, or mixed Gram-positive and Gram-negative bacteria, or an unknown pathogen. (**B**) GSE46914 [30]: PBMCs isolated from healthy donors: unstimulated (medium), stimulated once with LPS (LPS unprimed) or twice with LPS (LPS primed). Boxplot: Depicted are median and interquartile range (IQR, 25–75% percentile). A two-sided Mann–Whitney t-test was used to compare PAS across groups within the dataset. *p*-values (Bonferroni corrected) are indicated in the figures as ** *p* < 0.01 or ns (not significant). Pathway activity score (PAS) on Y-axis in log2odds.

AR PAS was increased in PBMCs from sepsis patients (n = 54) infected by Gram-positive, but not Gram-negative bacteria, when compared to healthy subjects (n = 16) (Figure 1A, GSE9960). No differences between healthy and sepsis patients were found for the activity of other STPs.

### 3.2. STP Activity in an In Vitro Gram-Negative Sepsis Model, PBMCs

Incubation of PBMCs with LPS has been proposed as a model for Gram-negative sepsis. We analyzed whether LPS modifies STP activity in PBMCs in vitro (GSE46914) (Figure 1B). PBMCs from healthy donors (n = 6) had been stimulated in vitro with LPS, either once (“LPS unprimed”) or twice LPS (“LPS primed”, 2 ng/mL followed by 100 ng/mL). In both conditions, LPS did not change STP activity, in line with the STP analysis results of the clinical sepsis study.

Thus, exposure of PBMCs to LPS, either in vivo during Gram-negative sepsis, or in vitro did not result in changes in STP activity.

### 3.3. STP Activity in an In Vitro Gram-Negative Sepsis Model—Whole Blood

**Figure 2:**
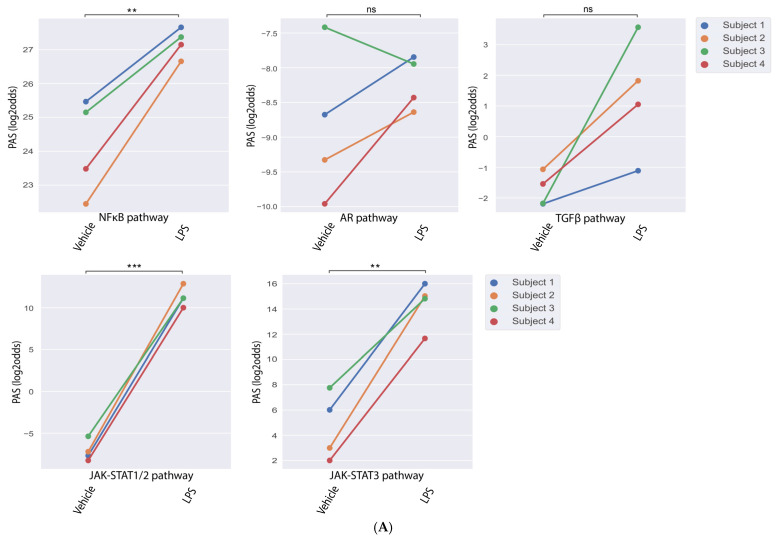

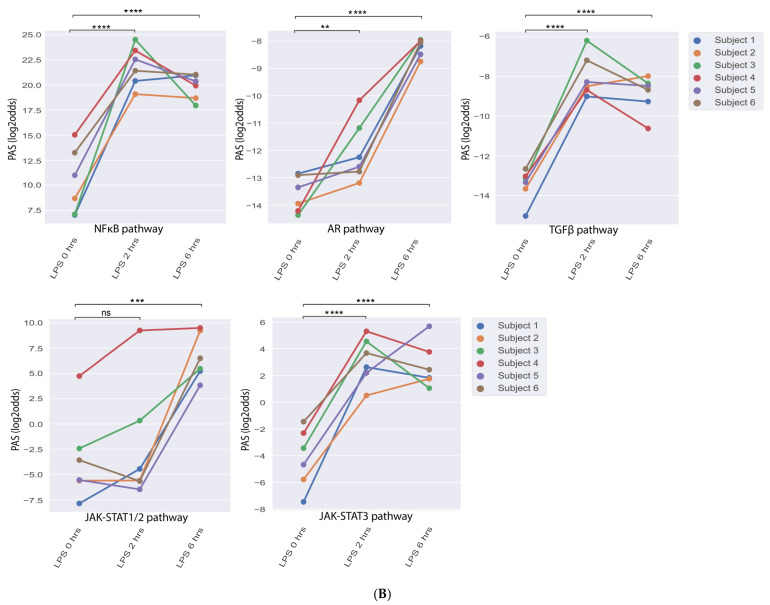
Pathway activity scores of whole blood from in vitro and in vivo endotoxemia LPS-based models. (**A**) GSE20114 [29]: Whole blood in vitro LPS-based model. Samples were stimulated for 4 h with LPS or vehicle. (**B**) GSE3284 [31]: Whole blood in vivo LPS endotoxemia model. Healthy humans received LPS. Whole blood was collected before LPS administration and 2 and 6 h thereafter. (**A**) Pathway activity score (PAS) of each individual patient is connected by a line and tested with a paired two-sided method. (**B**) A linear mixed-effects model was used to compare PAS across groups, and *p*-value annotations were added to the plot. *p*-values are indicated in the figures as ** *p* < 0.01, *** *p* < 0.001, **** *p* < 0.0001, or ns (not significant). PAS on Y-axis in log2odds.

Considering that patients with (Gram-positive and Gram-negative) sepsis displayed altered STP activity in whole blood [17], we analyzed data from two whole blood LPS sepsis models, one in vitro (GSE20114) and one in vivo model (GSE3284) (Figure 2). LPS stimulation (4 h, 10 µg/mL) of whole blood samples in vitro (n = 4) resulted in increased NFκB, JAK-STAT1/2, and JAK-STAT3 PAS (Figure 2A).

### 3.4. STP Activity in an In Vivo Gram-Negative Sepsis Model—Whole Blood

The human in vivo endotoxemia model has been frequently used to study mechanisms underlying inflammatory responses detected in sepsis, and to evaluate novel (pharmaceutical) interventions [7]. Intravenous injection of LPS elicited increases in NFκB, AR, TGFβ, and JAK-STAT3 PAS in whole blood samples after 2 h, followed by increased JAK-STAT1/2 PAS after 6 h (Figure 2B).

### 3.5. Differences in STP Activity Between Sepsis Models

There were differences in STP activity between in vivo and in vitro LPS-based sepsis models. The main difference was an increase in AR and TGFβ STP activity in the in vivo whole blood LPS model, not seen in the in vitro model.

With respect to absolute STP activity scores, another difference between in vitro and in vivo models became evident (Table 1). Compared to control samples of sepsis patients (PBMCs) and healthy subjects, control samples in the in vitro studies had higher mean NFκB, AR, and JAK-STAT3 PAS (both in PBMCs and whole blood) and higher TGFβ PAS (in whole blood). Such differences in absolute STP activity scores may be explained by sample handling and the fact that control samples for in vitro studies had been incubated with culture medium.

### 3.6. Comparison of STP Analysis with Conventional Transcriptome Data Analysis

In our study, we demonstrate the ability to extract valuable biological insights from transcriptome data using our STP analysis method. STP analysis results were compared with the data analysis results reported in the publications associated with the public datasets [28,29,30,31] (see Supplementary Materials). In these publications [28,29,30,31], conventional publicly available transcriptome analysis tools were used to analyze transcriptome data and make comparisons between sample groups. Comparisons revealed group-associated gene profiles, associated with potential differential involvement of various cellular processes. No information on the activity of STPs was reported. Unlike the transcriptome analysis tools used in these publications, our STP analysis focuses on quantitatively measuring STP activities in individual samples, offering unprecedented insights into sepsis laboratory models and the pathophysiology of clinical sepsis.

## 4. Discussion

In summary, we found that PBMCs from sepsis patients and from the in vitro sepsis model shared a similar STP activity profile, except for increased AR STP activity in Gram-positive sepsis patients. In whole blood of sepsis patients, we previously reported abnormally active AR and TGFβ and a trend towards increased activity of NFκB and JAK-STAT3 STPs [17]. In whole blood sepsis models, increased activity of NFκB, JAK-STAT1/2, and JAK-STAT3 STPs was found, while in vivo administration of LPS also increased AR and TGFβ STP activity. This was quite similar to what happened in sepsis patients, except for increased JAK-STAT1/2 STP activity, which only occurred in the sepsis models.

In PBMCs of sepsis patients, abnormally active STP activity was limited to the AR STP, and only in patients with Gram-positive bacterial infections. We previously reported that activity of the AR STP was increased in whole blood of Gram-positive and Gram-negative sepsis patients [17]. These results suggest that infection with Gram-negative bacteria, leads to increased activation of the AR pathway in whole blood, while Gram-positive infection-related pathogen-associated molecular patterns, such as lipoteichoic acid, may induce AR STP activity in both whole blood and PBMCs of sepsis patients. PBMCs lack the neutrophil fraction that is present in whole blood samples. This provides a likely explanation for our finding that abnormal STP activity was only detected in whole blood samples of sepsis patients and not in PBMCs. Neutrophils are known to play an important pathogenic role in sepsis [1,32]. Indeed, we reported before that activation of neutrophils with LPS results in increased JAK-STAT1/2, NFκB, TGFβ, and JAK-STAT3 STP activity [25]. These STPs are well known to play a role in the inflammatory response [33,34,35,36]. However, in LPS-stimulated neutrophils, activity of the AR STP did not increase [25]. Therefore, the increase in AR STP activity in whole blood from Gram-negative sepsis patients and from LPS-treated individuals seems to be a specific in vivo finding, probably requiring the systemic environment.

An intriguing observation was the large increase in JAK-STAT1/2 and NFκB STP activity in the in vitro and in vivo whole blood models for Gram-negative sepsis; this was not seen in sepsis patients. Differences in STP activities between LPS challenged volunteers and sepsis patients could be related to the fact that intravenously administered LPS elicits an acute inflammatory response, mainly driven by monocytes and neutrophils [7], whereas in sepsis patients responses are more sustained and also affect other cell types [1]. Indeed, sepsis patients have a clear T helper 2 (Th2) response and impaired Th1 response [37]. Earlier STAP-STP analysis of CD4+ T-cells showed lower JAK-STAT1/2 and NFκB PAS in Th2 cells, compared to Th1 cells [25]. Moreover, exposure of CD4+ T-cells to immunosuppressive supernatant of cancer cells resulted in lower JAK-STAT1/2 and NFκB STP activity [18]. We hypothesize that decreased JAK-STAT1/2 and NFκB STP activity in whole blood of sepsis patients reflects upregulation of an immunosuppressive CD4+ T cell population such as Th2 cells, as has been suggested before [30,38,39].

AR and TGFβ STP activity appeared to be increased in the in vivo whole blood model (human endotoxemia) and in patients with sepsis [17], but not in the in vitro whole blood model. This discrepancy is likely related to systemic factors, such as stress. Both the human endotoxemia model and clinical sepsis are associated with a strong stress response, resulting in elevated cortisol concentrations [40,41,42]. AR signaling and transcription of AR target genes can be mimicked by cortisol [43]. Indeed, both GR and AR signaling are known to be immunosuppressive in sepsis [17,44]. With respect to the TGFβ STP, it is relevant that neutrophils have the highest TGFβ STP activity compared to other immune cell types and their percentage increases during sepsis [25]. This implies that the increased TGFβ STP activity in the in vivo endotoxemia model may have been caused by a higher neutrophil count, induced by in vivo administration of LPS, while LPS cannot induce such a change when added in vitro.

We noted that the absolute activity scores of some STPs were higher in baseline samples from in vitro models compared to the samples that were directly processed after blood withdrawal. This might explain the non-significant increase in AR and TGFβ STP activity in the in vitro whole blood model. Likely causes are differences in sample collection, storage, and in vitro incubation in culture medium [45], [unpublished observations]. PAXgene tubes for in vivo blood collection contain RNA stabilizers to prevent post-collection changes in RNA expression. In agreement with this, STP activity in whole blood from healthy controls was comparable with baseline samples from the in vivo endotoxemia model [17]. While we do not know whether other collection methods change STP activity, the results support the use of PAX gene tubes to obtain reproducible RNA analysis results.

The whole blood in vivo endotoxemia model was most representative for sepsis patients, although this model represents the acute inflammation response rather than the Th2/immunosuppressive response seen in real sepsis patients [18]. However, this human endotoxemia model has limitations, such as ethical restrictions, recruitment problems, and subject heterogeneity reducing reproducibility. Furthermore, human endotoxemia models are unsuitable for drug screening. Therefore, the in vitro whole blood model is a better option for drug discovery. Sepsis models that insufficiently mimic sepsis patients provide a hurdle for drug development. For the development of adequate patient-representative sepsis models, it is essential to better characterize the dysfunction of specific immune cell (sub)types and their causal role in the pathogenesis of sepsis. STP analysis provides a valuable new tool to do this. Since drugs act on STPs, information on abnormally active STPs in specific immune cell types will help in drug development.

Using STAP-STP analysis technology, already existing in vitro sepsis models may be improved to better mimic patients with sepsis. Potential adjustments include adding or removing components from the culture medium, introducing other bacterial components, or adjusting incubation times, followed by measuring the STP activity profile to assess the induced change. For the two in vitro models, different LPS incubation protocols were used (different concentration and incubation time), which may have impacted STP activity. Because whole blood cannot be directly compared with PBMCs, currently available data do not enable us to pinpoint the effect of LPS concentration on STP activity in in vitro models. In sepsis patients, the LPS concentration in plasma has been described to lie around 500 pg/mL [46]. Experiments with the THP-1 monocytic cell line support the idea that low LPS concentrations can activate STPs, i.e., 0.5 ng/mL of LPS was sufficient to activate the AR, TGFβ, NFκB, and JAK-STAT1/2 STPs (our unpublished results, JAK-STAT3 STP activity not measured).

## 5. Limitations of the Study

Our study has limitations. We investigated cell mixtures, limiting our understanding of the specific roles of STPs in immune cell subsets in sepsis patients and laboratory sepsis models. For example, STP activity changes in low abundant immune cell types present in whole blood are likely missed in the overall STP activity score of the mixed cell population. Our analysis was also limited by the availability of datasets suitable for STAP-STP analysis. Comparison between sepsis models and patients was limited to LPS-based models, mimicking Gram-negative bacterial infections; data enabling this comparison for Gram-positive sepsis were not available. In the future, characterizing immune cell subsets with respect to STP activity is essential to unravel their distinct roles in sepsis and further improve laboratory models.

The STAP-STP technology enabled, for the first time, a quantitative phenotypic comparison between samples from sepsis patients and laboratory model systems. This is not possible with conventional transcriptome analysis tools such as Gene Set Enrichment Analysis (GSEA). Study insights can be utilized to select and optimize laboratory model systems for sepsis.

## Figures and Tables

**Table 1:** Pathway activity scores (mean and SD) for untreated control groups in vitro and in vivo.

		NFκB		AR		TGFβ		JAK-STAT1/2		JAK-STAT3	
Datasets	Baseline Group	Mean	SD	Mean	SD	Mean	SD	Mean	SD	Mean	SD
GSE46914	in vitro—PBMCs—medium control	17.44	5.32	−6.99	0.27	−12.27	3.59	−6.91	1.74	5.26	4.73
GSE9960	in vivo—PBMCs—control subjects	6.64	4.31	−8.72	0.98	−11.68	2.72	−5.52	4.11	−1.81	2.23
GSE20114	in vitro—whole blood—vehicle control	24.14	1.42	−8.85	1.09	−1.75	0.54	−7.14	1.25	4.69	2.66
GSE3284	in vivo—whole blood—0 hrs	10.36	3.31	−13.61	0.66	−13.48	0.83	−3.38	4.40	−4.19	2.24

Green—PBMCs samples. Orange—whole blood samples.

## Data Availability

The original contributions presented in the study are included in the article information, further inquiries can be directed to the corresponding author. The datasets analyzed during the current study are available in the GEO database repository, https://www.ncbi.nlm.nih.gov/geo/ (accessed on 1 September 2024).

## Supplementary Materials

The following supporting information can be downloaded at: https://www.mdpi.com/article/10.3390/cells14040311/s1, Supplementary Figure S1: Knowledge-based Bayesian computational signal transduction pathway model; Supplementary Figure S2: Characteristics of the patients from dataset GSE9960; Supplementary Materials: Comparison of Affymetrix data reported in the source publications and the results of STP analyses.
