# Supplementary material for: Comparison Between Signal Transduction Pathway Activity in Blood Cells of Sepsis Patients and Laboratory Models
Source: Cells. 2025 Feb 19;14(4):311. doi: 10.3390/cells14040311 (PMC11854017; doi:10.3390/cells14040311)
Supplement: Supplementary file 1 [file cells-14-00311-s001.zip › cells-3244070-supplementary.pdf]

Supplementary information

Supplementary Figure:

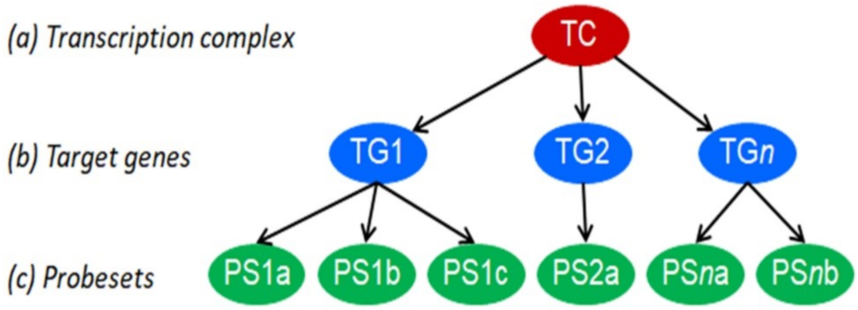

Figure S1: *Knowledge-based Bayesian computational signal transduction pathway model*. The network structure is used as basis for our modelling approach, consisting of three types of nodes: a) transcription factor complex (TC), b) target gene (TG), and c) microarray probe sets (PS) corresponding to target genes. Figure adapted from [13] with permission.

	All Patients (n = 70)	Training Set (n = 35)	Validation Set (n = 35)	*p*
Demographics				
Age (yr)	65.5 ± 18.4	62.0 ± 19.1	70.0 ± 17.7	0.39
Male/female	43/27	20/15	23/12	0.62
Comorbidities (%)				
Hypertension	17.1	20.0	14.3	0.75
Heart disease	14.3	8.6	20.0	0.31
Diabetes	12.9	17.1	8.6	0.48
COPD	11.4	8.6	14.3	0.71
Cancer	11.4	5.7	17.1	0.26
Trauma	17.1	8.6	11.4	0.71
Recent surgery (within last 7 days)	37.1	22.9	14.3	0.54
Severity of disease				
Mortality (%)	28.6	20.0	37.1	0.19
APACHE II	18.5 ± 7.2	20.0 ± 6.4	18.0 ± 8.1	0.99
Treatment (%)				
Mechanical ventilation	54.3	65.7	42.9	0.09
Renal dialysis	25.7	20.0	5.7	0.15
Vasopressor therapy	44.3	40.0	48.6	0.63
SIRS/sepsis (%)				
SIRS only	34.3	28.6	40.0	0.45
Sepsis	14.3	5.7	22.9	0.08
Severe sepsis/septic shock	51.4	65.7	37.1	0.03
Gram positive infection (%)	24.3	28.6	20.0	0.58
Gram negative infection (%)	27.1	25.7	28.6	1.00
Mixed infection (%)	14.3	17.1	11.4	0.73
Site of infection (%)				
Lung	18.6	14.3	22.9	0.54
Abdomen	10.0	11.4	8.6	1.00
Urinary tract	34.3	25.7	8.6	0.11
Others	37.1	51.4	59.9	0.63

Figure S2*: Characteristics of the patients from dataset GSE9960. Figure from* [28].

Supplementary Materials:

Comparison of Affymetrix data reported in the source publications and the results of STP analyses

1. *GSE20114*

Data analysis: differential gene expression, no pathway analysis.

No signaling pathway activity identified.

No comparison of model towards sepsis patients

2. *GSE3284*

Data analysis: differential gene expression, pathway analysis based on The Ingenuity Pathways Knowledge Base and interactome. several transcription factors were increased in the endotoxin model; NFKB, STAT, CREB, CEBP, SOCS3 and IKBK genes. Nine groups were identified based on differential gene expression using a knowledge-based network analysis:

Group 1, mitochondrial respiratory chain complex I (NDUF genes). Group 2, mitochondrial respiratory chain complex III (UQCR genes). Group 3, ATP synthase complex (ATP5 genes). Group 4, pyruvate dehydrogenase complex. Group 5, mitochondrial permeability transition pore complex. Group 6, elongation initiation factor complex (EIF3 genes). Group 7, ribosomal proteins (RPL, RPS genes). Group 8, COP9 signallosome (COPS genes). Group 9, proteasome (PSM genes).

No pathway activity analysis. NFkB and STAT differential gene expression relate to increased NFkB and JAK-STAT STP activity.

Comparison LPS model to patient: The authors indicate that some processes show similarities, but do not specify specific pathway activities. They further indicate that confirmation of results is needed in sepsis patients.

4. *GSE46914*

Data analysis: Differential expression, TRIF pathway and lectin complement pathway mentioned.

No signaling pathway activity identified.

Comparison model to patient, cited from publication: "The monocyte side of immunosuppression can be partly mimicked in-vitro by an endotoxin tolerance model."

5. GSE9960

Data analysis: Differential expression, No pathway activity analysis.

Conclusions cited from publication: “Our study did not identify any differentially expressed genes between Gram-positive and Gram-negative sepsis samples.”
